# Characterization of Plant-Based Meat Treated with Hot Air and Microwave Heating

**DOI:** 10.3390/foods13172697

**Published:** 2024-08-26

**Authors:** Chonnikarn Srikanlaya, Nantawan Therdthai

**Affiliations:** Department of Product Development, Faculty of Agro-Industry, Kasetsart University, Bangkok 10900, Thailand; chonnikarn.srik@ku.th

**Keywords:** microwave, baking, gluten, soy protein isolate, plant-based meat

## Abstract

Plant-based meat is growing globally due to health, environmental, and animal welfare concerns, though there is a need for quality improvements. This study assessed how different ratios of wheat gluten (WG) to soy protein isolate (SPI) and various baking methods—hot air (HA), microwave (MW), and a combination of both (HA–MW)—affect the physicochemical properties of plant-based meat. Increasing the SPI from 0% to 40% significantly enhanced lightness, hardness, chewiness, water-holding capacity, moisture content, and lysine (an essential amino acid) (*p* ≤ 0.05). Hardness and chewiness ranged from 4.23 ± 1.19 N to 25.90 ± 2.90 N and 3.44 ± 0.94 N to 18.71 ± 1.85 N, respectively. Baking methods did not affect amino acid profiles. Compared to HA baking, MW and HA–MW baking increased lysine content (561.58–1132.50 mg/100 g and 544.85–1088.50 mg/100 g, respectively) while reducing fat and carbohydrates. These findings suggest that a 40% SPI and 60% WG ratio with microwave baking (360 W for 1 min) optimizes plant-based meat, offering benefits to both consumers and the food industry in terms of health and sustainability.

## 1. Introduction

Plant-based meat diets are popular among vegan, vegetarian, and flexitarian consumers. The market for plant-based meat is projected to grow from $4.6 billion in 2018 to $85 billion by 2030, driven by health concerns, environmental sustainability, and animal welfare [1,2]. The share of people who follow a vegetarian diet worldwide includes 19% of the population in Asia, 16% in Africa, 8% in South America, 6% in Central America, and 6% in North America [3]. In terms of economic importance, plant-based meat has transitioned from a niche market to a mainstream one since 2015, with more than 6485 new product launches worldwide, indicating increased consumption of plant-based meat [4]. Additionally, plant-based meat has a 13%–26% lower impact on climate and greenhouse gas emissions compared to traditional meat production, which accounts for 80% of total greenhouse gas emissions [5]. The average greenhouse gas emissions for the production of 100 g of beef are 17.8 kg CO_2_, while those for 100 g of soy-based or wheat/soy-based meat products are only 0.21 kg CO_2_ and 0.23 kg CO_2_, respectively [6]. Improving the sensory quality of plant-based meat is quite challenging due to its often-perceived dry texture, off-flavors, and poor appearance. However, this is a key challenge for product developers to improve its texture, taste, aroma, and appearance in an economical and energy-efficient way by utilizing appropriate plant protein ingredients, techniques, and technologies [7]. Plant-based meat, or meat analogue, is a protein product derived from plants that provide low saturated fat, and it is cholesterol-free [3,4]. Wheat gluten and soy protein are commonly used to replicate animal proteins due to their cost-effectiveness and functional properties, including gelling, thickening, foaming, water binding, flavor binding, solubility, emulsification, and fibrous matrix formation [5,6,7].

Wheat gluten is rich in leucine and phenylalanine but lacks lysine, an essential amino acid. When hydrated, wheat gluten forms a cross-linked, three-dimensional network that mimics the fibrous structure of meat [8,9,10,11]. This network is created by disulfide bonds, which can be intramolecular or intermolecular depending on the protein type. Gluten proteins include gliadins and glutenins. Gliadins are low–to medium–molecular–weight monomeric proteins with primarily intramolecular disulfide bonds, while glutenins, with their mainly intermolecular disulfide bonds, provide the cohesive and viscoelastic properties needed for fibrous networks [12]. Consequently, wheat gluten has limited emulsion and foaming abilities [13].

Soy protein has a high molecular weight and surface hydrophobicity. Physical modification can alter its structure, leading to the formation of soy protein polymers [14]. Soy protein is primarily globulin, comprising 90% of its content, with glycinin (7S) and β-conglycinin (11S) as the main components [15,16]. These globular proteins are effective emulsifiers and foaming agents due to their water-soluble structures and non-polar regions, which facilitate adsorption at oil–water or air–water interfaces. During heating, unfolded globular proteins expose non-polar and sulfhydryl groups, promoting aggregation through hydrophobic interactions and disulfide bond formation. Generally, 11S globulins are more effective in foam stabilization, while 7S globulins excel as emulsifiers [14,17]. Soy protein is also rich in lysine, an essential amino acid often lacking in cereal proteins, as well as leucine and phenylalanine [11]. Thus, combinations of proteins (such as soy protein and wheat gluten) can enhance both nutritional value and functional properties, balancing amino acid profiles and creating a desirable fibrous structure [7].

Baking is a complex process involving simultaneous heat and mass transfer. During baking, physical, chemical, and biochemical changes occur, including starch gelatinization, protein denaturation, carbon dioxide release, volume expansion, water evaporation, crust formation, and browning reactions. Conventional baking methods, with their long baking times, can reduce the nutrient value of foods, such as vitamins and minerals. Recently, microwave baking has been proposed to improve product quality and processing efficiency [18,19,20,21]. It is also considered a sustainable and environmentally friendly technology [21]. Microwaves are electromagnetic waves with frequencies ranging from 300 MHz to 300 GHz [22]. During microwave treatment, ionic and dipolar particles migrate or rotate, generating heat [21]. For soy protein, microwaving causes protein molecules to expand and enhances molecular interactions, leading to cross-linking through both disulfide and non-disulfide covalent bonds [23]. Heat also induces protein aggregation and the formation of a three-dimensional network via disulfide bonds [17]. However, high energy absorption by polar protein groups can break disulfide and hydrogen bonds, causing protein unfolding and free radical formation. This exposure of hydrophobic groups enhances surface interactions [22,24]. Microwave treatment also alters the secondary structure of gel samples, demonstrating its potential for modifying and enhancing protein functionality in various applications [13].

As described above, wheat gluten provides the cohesive and viscoelastic properties needed to create fibrous networks that mimic the structure of meat but it lacks lysine, an essential amino acid for humans. Soy protein, which is rich in lysine, also contains globular proteins that are effective emulsifiers. Combining these components with a few minor ingredients is expected to produce plant-based meat that closely resembles real meat in both nutrition and texture. The characteristics of plant-based meat can vary based on the ratio of wheat gluten to soy protein and microwave heating conditions. However, there is limited information on the impact of microwave heating on plant-based meat. This study aims to investigate how different microwave baking conditions affect the physicochemical properties of plant-based meatballs made with a combination of wheat gluten and soy protein isolate. The findings will offer valuable insights for developing plant-based products, benefiting both the food industry and consumers by enhancing human health, protein sustainability, environmental sustainability, and animal welfare.

## 2. Materials and Methods

### 2.1. Materials

Wheat starch, vital wheat gluten (83.3% protein), and soy protein isolate (91.2% protein) were purchased from Thai Food and Chemical Co., Ltd., Bangkok, Thailand. Soybean oil (Sime Darby Oils Morakot Public Company Limited, Samut Prakan, Thailand) and all-in-one seasoning (F-plus, Nakhon Pathom, Thailand) were sourced from a local supermarket.

### 2.2. Preparation of Plant-Based Meat Using the Mechanical Elongation Method

Plant-based meat formulations (Table 1) with varying ratios of wheat gluten (WG) and soy protein isolate (SPI) were prepared using a modified method based on Chiang et al. [25] and Katayama and Wilson [23]. Briefly, wheat starch, wheat gluten, soy protein isolate, soy oil, and all-in-one seasoning (consisting of salt, sugar, mushroom powder, maltodextrin, and palm oil) were mixed with reverse osmosis water in a KitchenAid mixer (KitchenAid, MI, USA) at speed 1 for 5 min. The resulting dough was incubated at 40 °C for 60 min. It was then divided and processed in a Braun food processor (Braun, Kronberg, Germany) at speed 2 for 1 min and at speed 5 for 2 min to form a secondary dough. This dough was stretched to twice its initial length using a KitchenAid pasta extruder (KitchenAid, MI, USA).

Next, 20 g of the dough was placed in an open-top silicone mold (3.5 × 3.5 × 3.5 cm) and stored at 4 °C for 30 min before steaming for 11 min in an Otto King glass steamer (Otto King Glass Co. Ltd., Bangkok, Thailand). Finally, the dough was baked using one of the following methods: hot air baking (HA) at 160 °C for 10 min (oven: LG Electronics, Seoul, Korea), microwave baking at 360 W for 1 min (MW1) or 2 min (MW2), and hot air-microwave baking at 360 W at 160 °C for 1 min (HA–MW1) or 2 min (HA–MW2). The heating conditions were designed based on McClements et al. [26] with modifications, our previous findings [27,28], and the preliminary study.

### 2.3. Oil Holding Capacity Analysis

Oil holding capacity (OHC) was determined in triplicate following the method of Ketnawa et al. [8] with modifications. First, 0.5 g of each sample and 5 mL of canola oil were placed into a pre-weighed 15 mL centrifuge tube and mixed thoroughly using vortex shaking for 2 min. The mixture was then allowed to stand at room temperature for 30 min. After centrifugation at 3000× *g* for 30 min, the oil was discarded. The tube containing the residues was inverted for 10 min to drain excess oil, then reweighed. OHC was calculated using Equation (1) and expressed as grams of oil absorbed per gram of sample.

OHC [(g oil)/(g sample)] = (O_2_–O_1_–O_0_)/O_0_ (1)

where O_0_ = Weight of a ground sample, O_1_ = Weight of centrifuge tube, and O_2_ = Weight of centrifuge tube and residue.

### 2.4. Water Holding Capacity Analysis

Water holding capacity (WHC) was determined in triplicate according to Ketnawa et al. [8] with some modifications. First, 0.5 g of ground sample and 5 mL of deionized water were placed into a pre-weighed 15 mL centrifuge tube and mixed using vortex shaking for 2 min. The mixture was then allowed to stand at ambient temperature for 5 min. After centrifugation at 3000× *g* for 30 min, the supernatant was discarded. The tube containing the residues was inverted and left to stand for 5 min before reweighing. WHC was calculated using Equation (2) and expressed as grams of water absorbed per gram of sample.

WHC [(g H_2_O)/(g sample)] = (W_2_–W_1_–W_0_)/W_0_ (2)

where W_0_ = Weight of a ground sample, W_1_ = Weight of centrifuge tube, and W_2_ = Weight of centrifuge tube and residue.

### 2.5. Shear Force Analysis

The maximum shear force of analogues (10 × 10 × 20 mm) was measured in ten replications using a texture analyzer (TA–XT Plus, Stable Micro Systems, SRY, UK) equipped with a Warner–Bratzler (WB) shear blade with a 1 mm thick triangular slot cutting edge. The cut speed was set to 3 mm/s with 60% deformation according to Lorenzo et al. [29] with modifications.

### 2.6. Textural Profile Analysis

Hardness and chewiness of meat analogues (10 × 10 × 10 mm) were measured in ten replications using a texture analyzer (TA–XT Plus, Stable Micro Systems, SRY, UK) with a compression probe (P/75). The testing speed was set to 1 mm/s with 60% deformation according to Chiang et al. [25] with modifications.

### 2.7. Color Attributes

Color measurements were performed in triplicate following Yuliarti et al. [6] with modifications. The surface color of each sample was assessed at three different points from three meat analogues using a spectrophotometer (UltraScan PRO, HunterLab, VA, USA) with a 10° observer and D65 illuminant. Color parameters L*, a*, and b* were recorded, where L* represents lightness (0 = black, 100 = white), −a* indicates greenness, +a* indicates redness, −b* indicates blueness, and +b* indicates yellowness. Whiteness perception, derived from high lightness and low yellowness, was calculated using Equation (3) [8].

Whiteness = 100 − [(100 − L*)^2^ + a^2^ + b^2^]^1/2^ (3)

### 2.8. Nutritional Composition

Moisture, protein, fat, and ash contents of all samples were determined in duplicate using AOAC methods [30]. Total carbohydrate content was calculated by subtracting the sum of moisture, protein, fat, and ash from 100%. Soluble and insoluble dietary fibers were also measured in duplicate using AOAC methods [30].

### 2.9. Amino Acid Composition

The amino acid composition of all samples was measured in duplicate using high-performance liquid chromatography (HPLC) with standard amino acid references and reported as mg/100 g of sample [31]. The quantities of various amino acids were compared to a reference pattern of essential amino acids, which served as the standard for safe protein requirement. The reference values of the maintenance amino acid pattern, expressed as mg/g protein were as follows: 12 for histidine, 24 for isoleucine, 47 for leucine, 36 for lysine, 18 for sulfur amino acids, 30 for aromatic amino acids, 18 for threonine, 5 for trytophan, and 31 for valine [32]. The essential amino acid score (AAS) was calculated using Equation 4, as defined in the FAO/WHO/UNU report [33]:

4 $$\mathrm{AAS}=\frac{\mathrm{mg}\;\mathrm{of}\;\mathrm{amino}\;\mathrm{acid}\;\mathrm{in}\;1\;g\;\mathrm{test}\;\mathrm{protein}}{\mathrm{mg}\;\mathrm{of}\;\mathrm{amino}\;\mathrm{acid}\;\mathrm{in}\;\mathrm{requirement}\;\mathrm{pattern}}$$

### 2.10. Statistical Analysis

Experiments were designed using a completely randomized design (CRD) and were conducted under each experimental condition. Data were analyzed using one-way ANOVA to assess significant differences between treatments (*p* ≤ 0.05). Post hoc analysis was performed using Duncan’s multiple range test (*p* ≤ 0.05) with SPSS Statistics version 12.0.

## 3. Results and Discussion

### 3.1. Oil Holding Capacity and Water Holding Capacity of Plant-Based Meat

The OHC and WHC of samples substituted with soy protein isolate (SPI) and baked under various conditions are shown in Table 2. For the 0% SPI sample, the OHC with hot air (HA) baking was significantly higher compared to microwave (MW) and hot air-microwave (HA–MW) baking. The addition of SPI significantly enhanced OHC because the SPI’s hydrophobic groups interacted with oil droplets [14,17,34]. During extended heating in the HA oven, the SPI’s globular proteins unfolded, exposing hydrophobic regions that interacted with oil molecules [14,17,35]. In contrast, microwave baking might enhance hydrophilic sites on unfolded proteins, promoting hydrogen bonding between starch and protein molecules. Microwaves interacted with moisture in the sample, inducing rapid rotation and vibration of water molecules, which led to the unfolding of gluten proteins [36,37,38]. Longer microwave baking times might increase starch and protein aggregation, slightly decreasing OHC.

SPI contained both hydrophobic and hydrophilic characteristics. Samples with only wheat gluten (WG) had lower WHC compared to those containing both WG and SPI. Wheat proteins, due to their low solubility in water, tended to promote hydrophobic interactions that limit water binding and retention. Conversely, soy protein’s hydrophilic properties enhanced water binding and retention. In a WG–SPI composite system, the hydrophilic nature of soy protein improved WHC [13]. Şakıyan [39] also reported that wheat–soybean composite flour achieved greater WHC than wheat flour alone in cake-making, with higher soybean flour proportions enhancing WHC.

Baking conditions did not significantly affect the WHC of samples with 0% SPI. However, when SPI was added at 20–40%, MW and HA–MW baking resulted in lower WHC compared to HA baking. This was due to the SPI’s role in water retention and microwave energy absorption. The SPI helped retain water in the dough matrix and absorbed microwave energy, leading to faster heating. This increased the unfolding of proteins and aggregation, forming a denser and more compact structure, thereby reducing WHC [21].

Increasing MW and HA–MW baking times tended to improve WHC in WG-SPI samples due to increased dehydration. Significant improvement was observed only with 20% SPI addition. At 40% SPI, the effect of baking time on WHC was not significant, as the SPI’s influence on WHC enhancement dominated. Therefore, optimizing SPI content and baking conditions was crucial to achieving proper protein unfolding and aggregation, which allowed for better water retention.

### 3.2. Textural Properties and Shear Force Analysis of Plant-Based Meat

Table 3 shows the textural properties of samples substituted with SPI and baked under various conditions. The addition of SPI increased the hardness and chewiness of samples, ranging from 4.23 ± 1.19 to 25.90 ± 2.90 N for hardness and 3.44 ± 0.94 to 18.71 ± 1.85 N for chewiness. This increase was due to the inherent properties of SPI and WG and their interaction. Heating unfolded proteins and exposed hydrophobic regions that interacted and formed disulfide bonds, leading to protein aggregation and changes in texture strength. This aligned with Sukmanov et al. [40], who found that adding 2% and 4% SPI to pork batter significantly increased hardness and chewiness. Similarly, Youssef and Barbut [41] reported increased hardness and chewiness with 1.5% SPI addition. Hong et al. [42] also found that textured soy protein had higher hardness and chewiness compared to textured wheat gluten.

HA baking significantly affected the hardness, chewiness, and shear force of SPI-containing samples. HA baking enhanced hardness and chewiness but decreased shear force due to its slower heat distribution, which promoted starch gelatinization and protein denaturation. SPI formed firm, resilient gels, increasing hardness as SPI content rises [25].

For MW and HA–MW baking, 0% SPI samples exhibited lower hardness, chewiness, and shear force compared to HA baking. In contrast, 20% and 40% SPI samples showed higher hardness and chewiness with MW and HA–MW baking. This was likely because the SPI had a higher water-holding capacity than the WG, leading to faster heating and more protein aggregation [43]. Kutlu et al. [21] noted that MW samples had a tougher texture due to changes in gluten structure and inadequate gelatinization.

Hardness, chewiness, and shear force of MW samples with 20% and 40% SPI increased with longer baking times due to completed baking reactions like starch gelatinization and protein denaturation. Zheng et al. [24] reported that SPI exhibited higher viscoelasticity and a denser microstructure when heated by MW. In HA–MW samples with 20% SPI, shear force decreased with longer baking times, possibly due to the combination of surface heating from hot air and internal heating from the MW creating a denser protein matrix. At 40% SPI, shear force decreased with increased baking time, indicating that optimizing SPI–WG ratios and baking conditions was crucial for achieving the desired textural quality.

### 3.3. Color Attributes of Plant-Based Meat

Table 4 presents the color measurements (L*, a*, b*) and whiteness of control and experimental samples.

For HA baking, increasing SPI content (0%, 20%, and 40%) raised the L* value from 53.93 ± 1.14 to 56.41 ± 0.74 and whiteness from 47.92 ± 0.20 to 50.15 ± 0.67. These results were consistent with Li et al. [44], who observed similar color changes in pork myofibrillar proteins with varying SPI levels. Taikerd and Leelawat [43] also noted that SPI led to less browning compared to WG, attributing this to reduced browning reactions in SPI-containing meat analogues.

In contrast, MW and HA–MW baking resulted in lower L*, whiteness, and a* values for samples with 0% and 20% SPI compared to HA baking. MW baking altered the structure of starch and proteins, potentially making the samples more compact and affecting surface reflectance, which reduced L* and whiteness. Dong et al. [45] noted that the MW could also promote browning, similar to conventional methods, as observed in the study by Ye et al. [46], which found higher acrylamide and methylglyoxal levels in MW-baked potato chips compared to fried chips.

Increased baking time for MW and HA–MW samples resulted in further decreases in L*, whiteness, and a*. This reduction was attributed to harder textures and possible surface reflection changes due to extended baking times. Although water condensation during MW baking could hinder crust formation and Maillard browning, combining HA with MW baking improved surface temperature and browning [47].

**Figure 1:**
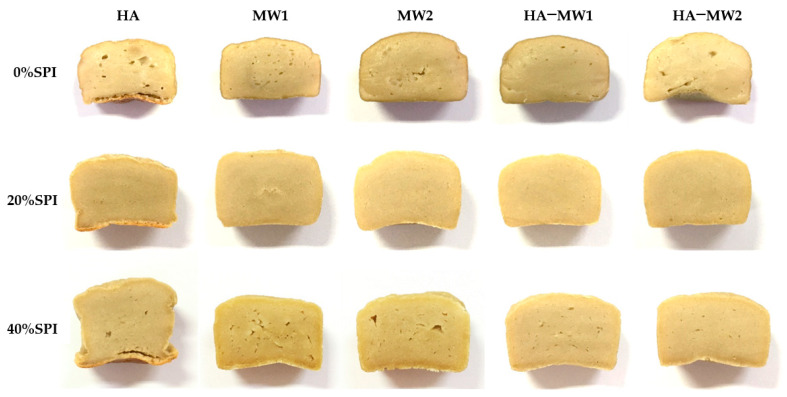
Photographs of plant-based meat showing variations in SPI content (0%, 20%, and 40%) and different baking conditions (hot air baking, microwave baking, and hot air-microwave baking).

For samples with 40% SPI, L* was relatively stable across baking conditions and times, and higher than the 0% and 20% SPI samples. Excessive SPI might contribute to natural color, and protein denaturation during baking could lead to incomplete browning due to free water migration (Figure 1).

### 3.4. Nutritional Composition of Plant-Based Meat

Table 5 presents the nutritional composition of plant-based meat samples, detailing moisture, protein, ash, lipid, and total carbohydrate contents. The proximate compositions varied based on the ratios of WG to SPI and the baking conditions used. The moisture content was lowest in the 100% WG plant-based meat. Adding SPI to the formula increased the moisture content, which ranged from 51.19 ± 0.0849% to 53.48 ± 0.0849%. This increase was attributed to WG’s relatively poor water-holding capacity (WHC) [25,48]. Protein content slightly increased with higher SPI ratios, ranging from 32.78 ± 0.2546% to 34.09 ± 0.0707%, compared to 32.85 ± 0.7000% in the 100% WG sample. Similarly, WG–SPI samples had higher ash content, ranging from 1.18 ± 0.0141% to 1.54 ± 0.0141%, compared to 0.78 ± 0.0212% in the 100% WG sample. This increase was due to the SPI’s higher protein content (96.10 ± 0.06% db) and ash content (3.52 ± 0.57%) compared to WG (82.76 ± 0.91% db, 0.93 ± 0.12%) [49]. Consequently, WG–SPI samples showed a decreased carbohydrate content, ranging from 8.50 ± 0.2767% to 9.29 ± 0.1833%, compared to 11.24 ± 1.5903% in the WG samples.

In terms of fat content, MW and HA–MW baking methods resulted in lower fat levels compared to HA baking. This reduction aligned with the decreased oil holding capacity (OHC) observed with microwave baking. The change in product structure, resulting from altered moisture distribution during microwave pretreatment, contributed to this outcome. Karacabey et al. [50] similarly reported that microwave-assisted drying as a pre-treatment before frying limited oil absorption.

The plant-based meat samples contained higher levels of insoluble dietary fiber compared to soluble dietary fiber. HA baking, especially with increased SPI content, enhanced both soluble and insoluble dietary fiber due to SPI’s higher fiber content compared to WG. Dietary fiber in soybean primarily consisted of cellulose, hemicellulose, and lignin [51], while wheat dietary fiber included non-starch polysaccharides and lignin, with arabinoxylans being the major fraction, constituting about 50% of wheat fiber [52]. Webb et al. [53] reported that extruded plant-based meat with 1.2% soy protein had higher fiber content compared to meat with 0.8% vital wheat gluten. Additionally, Nyaguthii et al. [54] found that increasing soy concentrate levels correlated with higher moisture and fiber content.

To enhance dietary fiber content in WG samples, the MW and HA–MW baking methods could be employed to induce amylose leaching from wheat starch granules. This process led to the recrystallization of amylose during cooling, resulting in increased levels of resistant starch, a type of dietary fiber [55]. Li et al. [44] demonstrated that MW treatment significantly increased the content of insoluble dietary fiber, soluble dietary fiber, and total dietary fiber in sorghum samples, with values rising to 3.72–7.45 g/100 g, 0.33–0.64 g/100 g, and 4.36–7.78 g/100 g, respectively. However, MW and HA–MW baking did not yield similar improvements in dietary fiber content for WG–SPI samples. This finding was consistent with the study by Svanberg et al. [56], which reported a decrease in total dietary fiber in green beans after MW treatment, primarily due to losses of soluble dietary fiber such as pectic polymers. The observed variations in the impact of microwave treatment on dietary fiber content were likely due to differences in the components and proportions of soy and wheat proteins, as well as the specific baking conditions used. Typically, the addition of SPI to samples improved water holding capacity which should, in theory, increase friction and mechanical energy during MW heating, leading to more amylose leaching and increased resistant starch. Nonetheless, SPI might interact with the leached amylose through hydrogen bonding, hydrophobic forces, and electrostatic adhesion [57], potentially reducing the formation of resistant starch and, consequently, the dietary fiber content.

### 3.5. Amino Acid of Plant-Based Meat

Table 6 presents the amino acid scores of all plant-based meat samples. An amino acid was considered limiting if its amino acid score (AAS) was lower than 1.0. For samples baked using hot air, increasing SPI content led to higher AAS for histidine (1.63 to 1.80), leucine (1.53 to 1.67), lysine (0.45 to 0.95), isoleutine (1.43 to 1.66), threonine (1.37 to 1.73), valine (1.19 to 1.34), and sulfur amino acids (3.19 to 3.96). Although samples initially lacked sufficient lysine (AAS lower than 1.0), the lysine score increased to close to 1.0 with higher SPI content. For microwave baked samples, the lysine content ranged from 0.47 to 0.98 for MW samples and from 0.43 to 0.93 for HA–MW samples. This finding was consistent with Chiang et al. [25], who observed a significant increase in lysine content when WG was replaced with SPI. This increase was attributed to WG’s lower lysine content compared to SPI.

For pork, AAS values were 2.93 for histidine, 2.07 for threonine, 2.05 for lysine, 1.39 for leucine, 1.59 for isoleucine, 1.78 for sulfur amino acids, 2.01 for aromatic amino acids, 1.31 for valine, and 2.17 for tryptophan [58]. For beef calf, the AAS ranged from 0.88 to 0.96 for sulfur amino acids, from 0.79 to 0.97 for aromatic amino acids, and from 0.89 to 0.99 for leucine [59]. Compared to traditional meat products, some ASS values for the plant-based samples were higher for leucine, isoleucine, sulfur amino acids, aromatic amino acids and valine. However, the AAS depended on the protein content of each product and the reference values for amino acid pattern. Although the plant-based meat with SPI contained lower lysine than the traditional pork, the consumption of soy protein, as a substitute for animal protein, has been associated with reduced blood sugar levels and lower obesity risks [60]. Therefore, it is still necessary to consume a combination of proteins or increase the intake of plant-based products to meet amino acid requirements [61].

In this study, microwave baking did not alter the types of amino acids present but did affect their quantities. The amino acid sequence of a protein polypeptide chain represented its primary structure, and heating primarily affects the protein’s spatial structure and conformation, rather than altering its amino acid sequence [62]. The changes in amino acid content after microwave treatment were attributed to cross-linking between lysine side chains and the involvement of ε-amino groups of arginine in the Maillard reaction [63,64].

**Figure 2:**
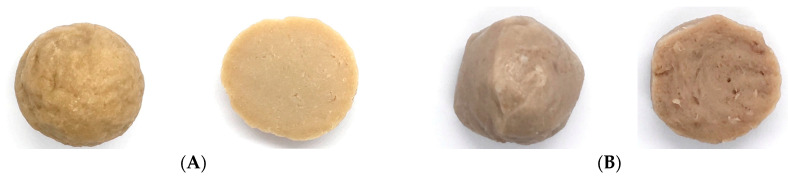
Photographs of the developed plant-based meatballs (**A**), compared to the regular meatballs (**B**).

According to comparative characteristics, quality, and nutrition relative to the original meatballs, the plant-based meatballs could be developed using a protein combination of 60% WG and 40% SPI and being microwaved at 360 W for 1 min (Figure 2).

## 4. Conclusions

This research provided a comparative evaluation of plant-based meat with varying wheat gluten (WG) and soy protein isolate (SPI) ratios under different microwave baking conditions. The substitution of SPI and the use of microwave-assisted baking significantly influenced several characteristics of the plant-based products, including hardness, chewiness, shear force, nutritional content, dietary fiber, water holding capacity (WHC), oil holding capacity (OHC), color, and amino acid scores (*p* ≤ 0.05). To achieve optimal plant-based meat quality, it was recommended to use a protein combination of 60% WG and 40% SPI, baked using a microwave (MW) at 360 W for 1 min. This specific formulation resulted in plant-based meatballs with the highest essential amino acid content, alongside lower fat and carbohydrate levels compared to other samples, while maintaining relatively low hardness. Although the developed plant-based meatballs were restricted to individuals who do not have celiac disease, due to the use of wheat gluten, the findings from this study provided valuable insights for developing new plant-based meat products. The use of microwave baking and the combination of WG and SPI offers benefits in terms of human health, protein sustainability, environmental sustainability, and animal welfare. Future research should explore alternative protein sources to expand the product’s appeal to a broader customer base.

## Figures and Tables

**Table 1:** Formulation of plant-based meats.

Sample	Baking Conditions		Ingredient (%w/w)					
			Wheat Gluten (WG, g)	Soy Protein Isolate (SPI, g)	Water (g)	Soybean Oil (g)	All-in-One Seasoning (g)	Wheat Starch (g)
100%WG + 0%SPI	HA	160 °C 10 min	41	0	55	2	1	1
	MW1	360 W 1 min	41	0	55	2	1	1
	MW2	360 W 2 min	41	0	55	2	1	1
	HA–MW1	160 °C 360 W 1 min	41	0	55	2	1	1
	HA–MW2	160 °C 360 W 2 min	41	0	55	2	1	1
80%WG + 20%SPI	HA	160 °C 10 min	32.8	8.2	55	2	1	1
	MW1	360 W 1 min	32.8	8.2	55	2	1	1
	MW2	360 W 2 min	32.8	8.2	55	2	1	1
	HA–MW1	160 °C 360 W 1 min	32.8	8.2	55	2	1	1
	HA–MW2	160 °C 360 W 2 min	32.8	8.2	55	2	1	1
60%WG + 40%SPI	HA	160 °C 10 min	24.6	16.4	55	2	1	1
	MW1	360 W 1 min	24.6	16.4	55	2	1	1
	MW2	360 W 2 min	24.6	16.4	55	2	1	1
	HA–MW1	160 °C 360 W 1 min	24.6	16.4	55	2	1	1
	HA–MW2	160 °C 360 W 2 min	24.6	16.4	55	2	1	1

Note: HA = Hot air oven at 160 °C for 10 min; MW1 = Microwave baking at 360 W for 1 min; MW2 = Microwave baking at 360 W for 2 min; HA–MW1 = Hot-air microwave baking at 360 W, 160 °C for 1 min; HA–MW2 = Hot-air microwave baking at 360 W, 160 °C for 2 min; WG = Wheat gluten; SPI = Soy protein isolate.

**Table 2:** Oil holding capacity (OHC) and water holding capacity (WHC) of plant-based meats.

Sample	Baking Conditions	OHC (g Oil/g Sample)	WHC (g H_2_O/g Sample)
100%WG + 0%SPI	HA	0.60 ± 0.16 ^d^	0.61 ± 0.06 ^f^
	MW1	0.57 ± 0.10 ^d^	0.62 ± 0.04 ^f^
	MW2	0.40 ± 0.10 ^e^	0.61 ± 0.11 ^f^
	HA–MW1	0.56 ± 0.10 ^d^	0.58 ± 0.07 ^f^
	HA–MW2	0.59 ± 0.08 ^d^	0.66 ± 0.04 ^f^
80%WG + 20%SPI	HA	0.83 ± 0.09 ^ab^	0.94 ± 0.14 ^d^
	MW1	0.77 ± 0.04 ^bc^	0.67 ± 0.07 ^f^
	MW2	0.83 ± 0.04 ^ab^	0.81 ± 0.07 ^e^
	HA–MW1	0.53 ± 0.12 ^de^	0.59 ± 0.02 ^f^
	HA–MW2	0.66 ± 0.11 ^cd^	0.69 ± 0.10 ^ef^
60%WG + 40%SPI	HA	0.97 ± 0.04 ^a^	1.46 ± 0.07 ^a^
	MW1	0.87 ± 0.02 ^ab^	1.30 ± 0.05 ^b^
	MW2	0.92 ± 0.03 ^ab^	1.33 ± 0.06 ^b^
	HA–MW1	0.83 ± 0.03 ^ab^	1.14 ± 0.08 ^c^
	HA–MW2	0.93 ± 0.01 ^a^	1.22 ± 0.06 ^bc^

Note: Values denoted by different superscripts within the column are significantly different (*p ≤* 0.05).

**Table 3:** Textural profiles of the plant-based meats.

Sample	Baking Conditions	Hardness (N)	Springiness (mm)	Chewiness (N)	Shear Force (N)
100%WG + 0%SPI	HA	8.17 ± 1.74 ^e^	0.95 ± 0.02 ^a^	6.75 ± 1.43 ^ef^	10.33 ± 0.54 ^cd^
	MW1	5.98 ± 1.64 ^ef^	0.96 ± 0.02 ^a^	4.99 ± 1.36 ^fg^	9.36 ± 0.68 ^e^
	MW2	4.23 ± 1.19 ^f^	0.94 ± 0.03 ^a^	3.44 ± 0.94 ^g^	10.30 ± 0.95 ^cd^
	HA–MW1	6.54 ± 2.52 ^ef^	0.95 ± 0.01 ^a^	5.30 ± 1.99 ^fg^	9.65 ± 0.27 ^e^
	HA–MW2	4.77 ± 0.98 ^f^	0.94 ± 0.02 ^a^	3.60 ± 1.41 ^g^	10.26 ± 0.56 ^d^
80%WG + 20%SPI	HA	10.74 ± 1.21 ^d^	0.93 ± 0.03 ^a^	8.46 ± 0.96 ^de^	9.33 ± 0.42 ^e^
	MW1	12.14 ± 2.39 ^cd^	0.94 ± 0.01 ^a^	9.18 ± 1.67 ^d^	11.21 ± 0.51 ^b^
	MW2	14.26 ± 1.05 ^c^	0.94 ± 0.01 ^a^	11.06 ± 0.84 ^c^	12.21 ± 1.07 ^a^
	HA–MW1	11.96 ± 2.28 ^cd^	0.94 ± 0.02 ^a^	8.98 ± 1.67 ^d^	10.87 ± 0.35 ^bc^
	HA–MW2	19.09 ± 1.72 ^b^	0.95 ± 0.01 ^a^	14.37 ± 1.16 ^b^	7.75 ± 0.52 ^g^
60%WG + 40%SPI	HA	24.46 ± 4.20 ^a^	0.94 ± 0.01 ^a^	18.26 ± 2.89 ^a^	7.63 ± 0.46 ^g^
	MW1	18.99 ± 3.86 ^b^	0.93 ± 0.03 ^a^	14.06 ± 2.80 ^b^	7.55 ± 0.87 ^hi^
	MW2	25.40 ± 6.07 ^a^	0.94 ± 0.01 ^a^	18.50 ± 4.03 ^a^	8.58 ± 0.84 ^g^
	HA–MW1	25.90 ± 2.90 ^a^	0.94 ± 0.01 ^a^	18.71 ± 1.85 ^a^	6.83 ± 0.25 ^h^
	HA–MW2	23.19 ± 4.38 ^a^	0.94 ± 0.01 ^a^	17.17 ± 3.30 ^a^	8.03 ± 0.51 ^fg^

Note: Values denoted by different superscripts within the column are significantly different (*p ≤* 0.05).

**Table 4:** Color of the outer layer of plant-based meats.

Sample	Baking Conditions	L*	a*	b*	Whiteness
100%WG + 0%SPI	HA	53.93 ± 1.14 ^cd^	3.24 ± 0.07 ^g^	21.89 ± 0.34 ^ef^	48.59 ± 0.90 ^cd^
	MW1	50.34 ± 0.99 ^fg^	3.49 ± 0.31 ^fg^	21.04 ± 0.91 ^f^	45.95 ± 1.03 ^fg^
	MW2	49.24 ± 0.17 ^gh^	4.10 ± 0.13 ^cd^	22.53 ± 0.88 ^de^	44.31 ± 0.25 ^h^
	HA–MW1	51.70 ± 0.69 ^ef^	3.53 ± 0.08 ^efg^	21.11 ± 0.64 ^f^	44.17 ± 0.67 ^ef^
	HA–MW2	48.28 ± 0.93 ^h^	3.86 ± 0.09 ^cdef^	21.10 ± 0.54 ^f^	44.01 ± 0.67 ^h^
80%WG + 20%SPI	HA	54.86 ± 0.24 ^bcd^	5.12 ± 0.15 ^ab^	25.47 ± 0.10 ^a^	47.92 ± 0.20 ^de^
	MW1	53.39 ± 0.15 ^de^	4.18 ± 0.31 ^cd^	23.79 ± 0.70 ^bc^	47.50 ± 0.35 ^de^
	MW2	51.26 ± 0.96 ^f^	5.27 ± 0.28 ^a^	25.26 ± 1.05 ^a^	44.84 ± 0.43 ^gh^
	HA–MW1	54.92 ± 0.81 ^bcd^	4.21 ± 0.45 ^c^	24.93 ± 0.22 ^ab^	48.31 ± 0.70 ^de^
	HA–MW2	50.03 ± 0.54 ^fgh^	4.99 ± 0.21 ^b^	22.86 ± 0.77 ^cde^	44.83 ± 0.61 ^gh^
60%WG + 40%SPI	HA	56.41 ± 0.74 ^ab^	3.78 ± 0.10 ^def^	23.89 ± 0.06 ^bc^	50.15 ± 0.67 ^bc^
	MW1	56.69 ± 0.51 ^ab^	3.93 ± 0.14 ^cde^	23.22 ± 0.10 ^cd^	50.70 ± 0.48 ^b^
	MW2	55.51 ± 2.02 ^bc^	3.69 ± 0.18 ^ef^	22.15 ± 0.96 ^def^	50.14 ± 1.47 ^bc^
	HA–MW1	56.59 ± 2.18 ^ab^	3.65 ± 0.07 ^ef^	22.43 ± 0.74 ^de^	50.98 ± 1.63 ^ab^
	HA–MW2	57.96 ± 0.95 ^b^	3.22 ± 0.12 ^g^	22.46 ± 0.50 ^de^	52.22 ± 0.66 ^a^

Note: Values denoted by different superscripts within the column are significantly different (*p* ≤ 0.05). L* = Lightness; −a* = Greenness; +a* = Redness, −b* = Blueness; +b* = Yellowness.

**Table 5:** Nutritional composition of plant-based meats.

Sample	Baking Conditions	Moisture Content (%)	Protein (%)	Fat (%)	Carbohydrates (%)	Ash (%)	Total Dietary Fiber g/100 g	Soluble Dietary Fiber g/100 g	Insoluble Dietary Fiber g/100 g
100%WG + 0%SPI	HA	51.59 ± 0.86 ^fg^	32.85 ± 0.70 ^bcd^	3.55 ± 0.05 ^f^	11.24 ± 1.59 ^f^	0.78 ± 0.02 ^b^	2.69 ± 0.23 ^i^	0.00 ± 0.00 ^g^	2.69 ± 0.23 ^f^
	MW1	52.89 ± 1.09 ^ef^	33.15 ± 0.03 ^cd^	2.74 ± 0.04 ^l^	10.46 ± 1.01 ^f^	0.77 ± 0.01 ^b^	7.92 ± 0.16 ^b^	1.63 ± 0.31 ^c^	6.29 ± 0.47 ^a^
	MW2	51.34 ± 0.51 ^fg^	32.44 ± 0.74 ^d^	2.83 ± 0.06 ^k^	12.61 ± 0.19 ^f^	0.79 ± 0.02 ^a^	4.38 ± 0.01 ^g^	1.09 ± 0.06 ^de^	3.29 ± 0.07 ^ef^
	HA–MW1	52.64 ± 1.06 ^de^	32.15 ± 1.07 ^d^	3.03 ± 0.07 ^j^	11.39 ± 0.07 ^f^	0.79 ± 0.01 ^b^	6.19 ± 0.57 ^d^	1.66 ± 0.04 ^c^	4.53 ± 0.61 ^b^
	HA–MW2	51.08 ± 0.19 ^fg^	35.05 ± 0.93 ^a^	3.19 ± 0.10 ^i^	9.90 ± 0.88 ^f^	0.79 ± 0.04 ^bc^	7.05 ± 0.05 ^c^	2.62 ± 0.33 ^a^	4.43 ± 0.38 ^bc^
80%WG + 20%SPI	HA	51.19 ± 0.08 ^fg^	34.09 ± 0.07 ^bc^	4.25 ± 0.01 ^a^	9.29 ± 0.18 ^d^	1.18 ± 0.01 ^bcd^	5.32 ± 0.17 ^ef^	0.86 ± 0.05 ^e^	4.47 ± 0.22 ^b^
	MW1	53.00 ± 0.07 ^bcde^	33.21 ± 0.48 ^bc^	3.85 ± 0.03 ^c^	8.83 ± 0.37 ^e^	1.12 ± 0.01 ^de^	4.59 ± 0.03 ^g^	0.44 ± 0.02 f	4.15 ± 0.01 ^bcd^
	MW2	52.92 ± 0.16 ^cde^	32.66 ± 0.08 ^cd^	4.02 ± 0.03 ^b^	9.25 ± 0.26 ^de^	1.16 ± 0.01 ^cde^	3.43 ± 0.41 ^h^	0.06 ± 0.00 ^g^	3.37 ± 0.40 ^ef^
	HA–MW1	53.07 ± 0.11 ^bcde^	34.33 ± 0.59 ^ab^	3.82 ± 0.01 ^cd^	7.64 ± 0.70 ^e^	1.15 ± 0.01 ^e^	4.59 ± 0.17 ^g^	1.19 ± 0.11 ^d^	3.40 ± 0.06 ^def^
	HA–MW2	51.36 ± 0.17 ^fg^	34.68 ± 0.11 ^ab^	4.04 ± 0.01 ^b^	8.73 ± 0.06 ^e^	1.19 ± 0.01 ^cde^	5.90 ± 0.15 ^de^	1.29 ± 0.10 ^d^	4.60 ± 0.05 ^b^
60%WG + 40%SPI	HA	53.48 ± 0.08 ^bcd^	32.78 ± 0.25 ^cd^	3.70 ± 0.05 ^ef^	8.50 ± 0.28 ^c^	1.54 ± 0.01 ^cde^	8.96 ± 0.57 ^a^	2.33 ± 0.16 ^b^	6.63 ± 0.40 ^a^
	MW1	54.58 ± 0.07 ^a^	32.16 ± 0.02 ^d^	3.24 ± 0.03 ^hi^	8.51 ± 0.03 ^b^	1.52 ± 0.01 ^cde^	4.92 ± 0.17 ^fg^	1.35 ± 0.03 ^d^	3.57 ± 0.14 ^de^
	MW2	53.76 ± 0.06 ^b^	32.68 ± 0.15 ^cd^	3.30 ± 0.02 ^gh^	8.72 ± 0.18 ^b^	1.55 ± 0.01 ^de^	5.91 ± 0.19 ^de^	1.30 ± 0.05 ^d^	4.60 ± 0.14 ^b^
	HA–MW1	53.53 ± 0.32 ^bc^	32.39 ± 0.04 ^cd^	3.34 ± 0.01 ^g^	9.18 ± 0.25 ^b^	1.57 ± 0.04 ^cde^	4.60 ± 0.33 ^g^	1.04 ± 0.00 ^de^	3.56 ± 0.33 ^de^
	HA–MW2	51.39 ± 0.04 ^fg^	34.46 ± 0.06 ^ab^	3.69 ± 0.02 ^de^	8.83 ± 0.11 ^a^	1.64 ± 0.02 ^cde^	4.58 ± 0.47 ^g^	0.86 ± 0.02 ^e^	3.72 ± 0.49 ^cde^

Note: Values denoted by different superscripts within the column are significantly different (*p ≤* 0.05).

**Table 6:** Amino acid score (AAS) of plant-based meats.

Sample	Baking Conditions	Histidine	Leucine	Lysine	Isoleucine	Threonine	Tryptophan	Valine	AAA (Phe + Tyr)	SAA (Met + Cys)
100%WG + 0%SPI	HA	1.63	1.53	0.45	1.43	1.37	0.90	1.19	2.82	3.19
	MW1	1.64	1.54	0.47	1.45	1.38	0.86	1.20	2.83	3.29
	MW2	1.74	1.63	0.48	1.49	1.51	0.86	1.29	3.01	3.64
	HA–MW1	1.64	1.54	0.48	1.52	1.41	0.69	1.25	2.95	3.23
	HA–MW2	1.53	1.43	0.43	1.34	1.27	0.74	1.15	2.68	2.75
80%WG + 20%SPI	HA	1.72	1.58	0.74	1.35	1.61	0.66	1.20	2.95	3.34
	MW1	1.47	1.44	0.63	1.37	1.36	0.67	1.19	2.70	2.77
	MW2	1.44	1.35	0.54	1.33	1.32	0.66	1.13	2.50	2.47
	HA–MW1	1.49	1.47	0.61	1.42	1.41	0.62	1.18	2.67	3.37
	HA–MW2	1.60	1.47	0.64	1.40	1.45	0.63	1.15	2.67	3.55
60%WG + 40%SPI	HA	1.80	1.67	0.95	1.66	1.73	0.75	1.34	2.94	3.96
	MW1	1.83	1.69	0.98	1.79	1.67	0.71	1.43	2.98	3.99
	MW2	1.70	1.57	0.91	1.46	1.68	0.67	1.21	2.80	3.95
	HA–MW1	1.78	1.61	0.93	1.52	1.71	0.79	1.26	2.87	3.77
	HA–MW2	1.67	1.51	0.87	1.57	1.59	0.72	1.28	2.64	3.50

Note: AAA = Aromatic amino acids; SAA, = Sulfur amino acids; Phe = Phenylalanine; Tyr = Tyrosine; Met = Methionine; Cys = Cystine.

## Data Availability

The original contributions presented in the study are included in the article, further inquiries can be directed to the corresponding author.
